# Poor statistical reporting, inadequate data presentation and spin persist despite Journal awareness and updated *Information for Authors*

**DOI:** 10.12688/f1000research.142841.1

**Published:** 2023-11-20

**Authors:** Martin Héroux, Joanna Diong, Elizabeth Bye, Georgia Fisher, Lucy Robertson, Annie Butler, Simon Gandevia

**Affiliations:** 1 School of Biomedical Sciences, University of New South Wales, Sydney, New South Wales, 2052, Australia; 2 Neuroscience Research Australia, Sydney, NSW, 2031, Australia; 3 School of Biomedical Sciences, The University of Sydney, Sydney, New South Wales, 2006, Australia; 4 Faculty of Medicine, Health and Human Sciences, Macquarie University, Sydney, New South Wales, 2109, Australia; 5 School of Clinical Medicine, University of New South Wales, Sydney, New South Wales, 2031, Australia

**Keywords:** Meta-research, research quality, scientific reporting, reproducibility

## Abstract

Sound reporting of research results is fundamental to good science. Unfortunately, poor reporting is common and does not improve with editorial educational strategies. We investigated whether publicly highlighting poor reporting at a journal can lead to improved reporting practices. We also investigated whether reporting practices that are required or strongly encouraged in journal *Information for Authors* are enforced by journal editors and staff. A 2016 audit highlighted poor reporting practices in the Journal of Neurophysiology. In August 2016 and 2018, the American Physiological Society updated the *Information for Authors*, which included the introduction of several required or strongly encouraged reporting practices. We audited Journal of Neurophysiology papers published in 2019 and 2020 (downloaded through the library of the University of New South Wales) on reporting items selected from the 2016 audit, the newly introduced reporting practices, and items from previous audits. Summary statistics (means, counts) were used to summarize audit results. In total, 580 papers were audited. Compared to results from the 2016 audit, several reporting practices remained unchanged or worsened. For example, 60% of papers erroneously reported standard errors of the mean, 23% of papers included undefined measures of variability, 40% of papers failed to define a statistical threshold for their tests, and when present, 64% of papers with p-values between 0.05 and 0.1 misinterpreted them as statistical trends. As for the newly introduced reporting practices, required practices were consistently adhered to by 34 to 37% of papers, while strongly encouraged practices were consistently adhered to by 9 to 26% of papers. Adherence to the other audited reporting practices was comparable to our previous audits. Publicly highlighting poor reporting practices did little to improve research reporting. Similarly, requiring or strongly encouraging reporting practices was only partly effective. Although the present audit focused on a single journal, this is likely not an isolated case. Stronger, more strategic measures are required to improve poor research reporting.

## Introduction

Scientific discovery and the translation of findings into practice requires complete, transparent and unbiased reporting. Although fundamental to the scientific method, sound reporting practices are not always adhered to. Despite clear recommendations, ^1^^–^^11^ poor statistical reporting and biased interpretation of results are common. ^12^^–^^23^

In 2011, the Journal of Physiology and the British Journal of Pharmacology jointly published a series of editorials to educate their readership about statistics and scientific reporting. ^24^ These editorials covered topics such as statistical terminology and procedures, ^25^^,^^26^ sample size and statistical power, ^27^^,^^28^ and results presentation and interpretation. ^29^^–^^31^ Together, these editorials became reporting guidelines referenced in the *Information for Authors* of these journals. Unfortunately, an audit of papers published before and after these guidelines were implemented revealed no improvement in reporting practices. ^32^ For example, over 80% of audited papers inappropriately used the standard error of the mean (SEM) to summarise data variability, while 60% of papers with p-values between 0.05-0.1 misinterpreted these as statistical trends (i.e. spin: reporting results so that they are interpreted in a more favourable light). Thus, educational editorials and encouragement from journals are not sufficient to improve reporting practices.

In 2016, a Letter to the Editor called attention to the high prevalence of poor statistical reporting practices in the Journal of Neurophysiology. ^33^ This audit evaluated all papers published in 2015, and found that 65% of papers inappropriately used the SEM, only 58% of papers reported exact p-values (e.g. p=0.021 *versus* p < 0.05), and 57% of papers with p-values between 0.05-0.1 resorted to spin.

To enhance the quality of scientific reporting in its journals, the American Physiological Society revised its *Information for Authors* in August 2016 and June 2018. Several reporting practices were now required or strongly encouraged.

These events presented a unique opportunity. Specifically, we wondered whether publicly highlighting poor reporting practices would motivate a journal to crack down on poor reporting practices. Moreover, we wondered whether reporting practices that were required or strongly encouraged – not merely encouraged as in the case of the Journal of Physiology and the British Journal of Pharmacology – would result in improved reporting practices.

The present study addressed two research questions: 1) Do reporting practices improve when a journal is informed of poor reporting practices? 2) Do publications adhere to reporting practices that are either required or strongly encouraged? We audited papers published in the Journal of Neurophysiology in 2019 and 2020. Audit items included the three items from the original Journal of Neurophysiology audit, ^33^ two required and two strongly encouraged reporting practices from the updated American Physiological Society Information for Authors, and four items from other audits we have conducted. ^32^^,^^34^

## Methods

### Eligibility criteria

Original research papers published in the Journal of Neurophysiology in the years 2019 and 2020 were eligible for inclusion. Papers were excluded if they were editorials, reviews, errata, comments or rebuttals. Full-text PDF files of eligible papers were downloaded through the library of the University of New South Wales in January 2022.

### Audit items

**Items from the original audit**

We assessed whether one or more of the following measures were used to summarise the variability of data or the size of an effect: standard error of the mean (SEM), standard deviation (SD), 95% confidence interval (95% CI) or interquartile range (IQR). We also assessed whether any summary statistic was not defined; this typically happens when the ± symbol or errors bars on plots are used but are not defined. The *Guidelines for reporting statistics in journals published by the American Physiological Society,* ^1^ a document that is referenced in the Journal of Neurophysiology *Information for Authors*, states that the SEM should not be used to summarise the variability of data or the size of an effect.

Additionally, we assessed whether papers reported exact p-values consistently, inconsistently, or not at all. The use of exact p-values is recommended by the *Guidelines for reporting statistics in journals published by the American Physiological Society.* ^1^

Finally, we assessed whether authors interpreted p-values close to, but above the selected threshold for statistical significance, typically p=0.05, as statistically significant or statistical trends (i.e. spin). This practice is misleading: more often than not, if additional data are collected, these p-values increase. ^3^ To be able to compare our results to those of a more recent audit, ^34^ we also determined the prevalence of spin across all papers, regardless of whether or not such a p-value was present.

**Items required or strongly encouraged in updated *Information for Authors***

Although several new reporting practices were introduced in the 2016 and 2018 updates to the *Information for Authors* for American Physiological Society journals (underlying data: *Extract from Information for Authors*), we selected four items that were broadly relevant, easily assessed, and, in two cases, related to items from previous audits.

We audited two required reporting practices introduced in August 2016. First, we assessed whether the number of samples or animals that contributed to plotted results were specified in figures or figure legends. Second, if statistical results were reported in a figure or figure legend, we assessed whether the statistical test used was specified in the figure legend.

We also audited two strongly encouraged reporting practices introduced in August 2018. First, we assessed whether data underlying the results were made available upon request or in a public repository. Second, we assessed whether graphical data “showed the range of data points in relation to the mean value and significance (i.e. dot-whisker plots) rather than formats that may mask variability (i.e. bar graphs)” and was “formatted to best convey the variability in the results” (underlying data: *Extract from Information for Authors*). It is unfortunate that the *Information for Authors* specifically reference’dot-whisker plots’ given that a mean (dot) and a two-sided error bar (whisker) is not different from a bar graph. That is, a dot-whisker plot can mask variability just as much as a bar graph. Nevertheless, because this reporting practice was introduced to encourage authors to convey the variability of plotted data and results, we scored this item as’yes’ when figures met this requirement: if the individual data points used to generate the summary statistics were plotted (regardless of the type of summary statistic used), or if box-and-whisker (median, IQR, range) or violin plots were encouraged.

**Items from previous audits**

To broaden the scope of the present audit and to allow for direct comparison with our previous results, we selected four additional items from two previous audits. ^32^^,^^34^

We assessed whether a sample size calculation was performed prior to the start of the study. In confirmatory studies, it is important to determine *a priori* the sample size required to achieve sufficient statistical power or generate precise estimates of the investigated effects. ^4^^,^^35^^,^^36^

We also assessed whether the study protocol was registered prior to the start of the study. Study registration is one of the simplest ways to improve the rigour of a study; it combats the selective reporting of outcomes, the cherry-picking of results, and p-hacking. ^37^^–^^41^

Next, we assessed whether plots included individual data points. As indicated in the Journal of Neurophysiology *Information for Authors*, “the same bar graph can represent many different datasets” (underlying data: *Extract from Information for Authors*). Similarly, in their series of educational editorials published in the Journal of Physiology and the British Journal of Pharmacology, Drummond & Vowler ^30^ encourage researchers to “use methods of data presentation that allow inspection, not concealment, of the nature of the distribution of the data.” While closely related to the audit item on variability of plotted data and results, the present item was included to allow for a direct comparison with previous audit results.

Finally, we assessed if a probability threshold was defined when frequentist statistics were used. This recommendation comes from the *Guidelines for reporting statistics in journals published by the American Physiological Society* ^1^: “Define and justify a critical significance level appropriate to the goals of your study”.

### Scoring manual and pilot testing

A scoring manual was created to increase the consistency across investigators (extended data: *Scoring manual*). Next, five eligible papers were selected randomly and audited by all investigators. A consensus meeting was held and agreement across investigators was assessed. Wording of audit items and the scoring manual was refined to improve the consistency and accuracy of audit results.

### Data extraction and analysis

Each investigator was randomly allocated ∼ 80 papers to audit, which they assessed independently.

If investigators were unsure how to score an audit item for a given paper, it was discussed amongst the team and agreement was reached by consensus.

Data were summarised with counts and percentages of papers that fulfilled the scoring criteria for each audit item. As it was not directly relevant to the audit, risk of bias was not assessed. Data processing and analysis were performed in Python (v3.9). Raw data and summary results are provided (underlying data: *Audit Results*). Although this study is not a systematic review, PRISMA guidelines were adhered where possible ^42^ (extended Data: *PRISMA Checklists*).

### Statistical analyses

For audit items from the original audit of papers published in 2015 in the Journal of Neurophysiology, ^33^ we compared the proportions of responses for results from the current audit to those of the previous audit using the proportions_ztest function from the Python statsmodels package, ^43^ with the level of significance set at α =0.05.

## Results

**Figure 1.**
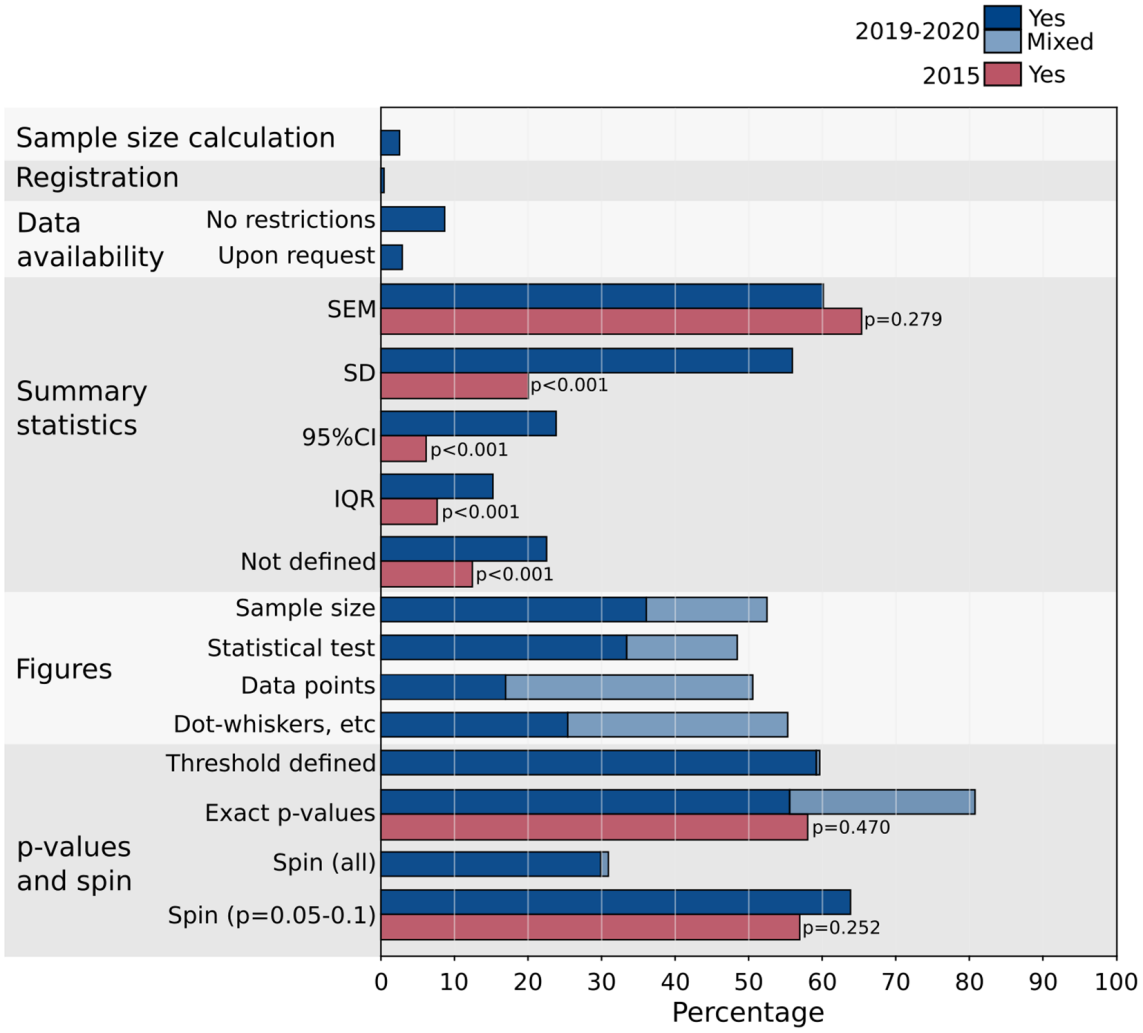
Audit results. The results of the present audit (2019-2020 papers) are plotted in blue. Values in light blue represent the percentage of papers that adhered to the reporting requirement, but not consistently. Also plotted are the results from the previous audit of 2015 Journal of Neurophysiology papers, including the results from the tests of proportions between the 2015 and 2019-2020 results. Detailed results are presented in Table 1. Standard error of the mean (SEM); interquartile range (IQR); standard deviation (SD); confidence interval (CI).

Summary results for the present audit and the 2015 audit are presented in Figure 1, including the statistical results of the proportions tests. Detailed results from the present and previous audits are presented in Table 1.

**Table 1.** Percentages and count data from current and previous audits.

			JNP -2020 current study	JNP Héroux 2016	JP and BJP 2007-2015 Diong *et al*. 2018	NeuRA 2017-2018 Héroux *et al*. 2022
Sample size calculation			2.7% (15/556)	—	—	13.3% (56/421)
Registered			0.5% (3/580)	—	—	6.2% (28/452)
Data availability ^†^	No restrictions		9.0% (52/580)	—	—	6.0% (27/452)
	Upon request		3.1% (18/580)	—	—	—
Summary statistics	SEM ^*^		60.2% (343/570)	65.4% (178/278)	80.0% (593/741) ^§^	32.4% (138/426)
	SD		56.0% (319/570)	20.2% (55/278)	—	—
	95% CI ^*^		23.9% (131/570)	6.2% (17/278)	—	—
	IQR		15.3% (87/570)	7.7% (21/278)	—	—
	Not defined		22.6% (129/570)	12.5% (34/278)	12.9% (97/750) ^§^	30.4% (129/424)
Figures	Sample size ^††^	Yes:	36.7% (205/559)	—	—	—
		Mixed:	16.5% (92/559)	—	—	—
	Statistical test ^††^	Yes:	33.7% (164/487)	—	—	—
		Mixed:	15.2% (85/487)	—	—	—
	Data points	Yes:	17.1% (85/497)	—	2.8% (10/356) ^¶^	—
		Mixed	33.8% (168/497)	—	—	—
	Dot-whisker, etc ^†^	Yes:	25.9% (128/497)	—	—	—
		Mixed	30.1% (149/497)	—	—	—
*p*-values and spin	Threshold defined ^*^	Yes:	59.3% (327/551)	—	58.4% (160/274)	62.3% (255/409)
		Mixed:	0.5% (3/551)	—	—	—
	Exact p-values ^*^	Yes:	55.7% (301/540)	58.4% (160/274)	7.4% (52/698) ^¶^	57.4% (231/402) ^¶^
		Mixed:	25.4% (137/540)	—	—	—
	Spin (all)	Yes:	30.0% (167/556)	—	—	31.1% (127/409)
		Mixed:	1.1% (6/556)	—	—	—
	Spin (p=0.05-0.1)		64.0% (171/267)	56.8% (42/74)	60.0% (36/60)	—

Journal of Neurophysiology (JNP); Journal of Physiology (JP); British Journal of Pharmacology (BJP); Neuroscience Research Australia (NeuRA); standard error of the mean (SEM); interquartile range (IQR); standard deviation (SD); confidence interval (CI).

* Indicates items that were encouraged or, for the SEM, discouraged.

^†^ Indicates items that were strongly encouraged.

^††^ Indicates items that were required.

^§^ Values pooled and averaged across years; applied to summary statistics reported in text and figures.

^¶^ Scored ‘yes’ if item always adhered to; mixed reporting scored as ‘no’.

In total, 580 original research papers published in 2019 and 2020 were audited. Few papers included a sample size calculation (2.7%) or indicated that the study protocol was registered prior to the start of the study (0.5%). Similarly, few papers made their data available publicly (9.0%) or upon request (3.1%), despite this practice being strongly encouraged by the updated American Physiological Society Information for Authors.

Overall, 60.2% of papers reported SEM, similar to the 65.4% noted in the original audit of 2015 papers. While the percentage of papers that reported SD, IQR, and 95% CI nearly doubled between 2015 and 2019-2020, so too did the percentage of papers that included undefined measures of variability (12.5% *vs* 22.6%).

Although it was a required reporting practice, only a third of papers consistently specified the type of statistical tests or the number of samples or animals in their figure legends. Similarly, despite being strongly recommended, only a third of papers consistently plotted their data in a manner that conveyed the variability of the data.

In line with results from two previous audits, ^32^^,^^34^ only 59.3% of papers defined a probability threshold. And finally, in line with results from the previous audit of 2015 papers, only 55.7% of papers consistently reported exact p-values, while 64.0% of papers with p-values between 0.05 and 0.1 reported them as statistical trends.

## Discussion

We provide evidence that publicly highlighting poor reporting practices in a journal does not lead to improvements. Moreover, our results indicate that strongly encouraging or requiring reporting practices does not guarantee these will be adhered to or enforced.

### Interpreting audit results

Compared to the original audit, the majority of poor reporting practices were unchanged or worsened. For example, ∼ 60% of audited papers reported SEM while ∼ 40% of papers failed to specify a threshold value for their statistical tests. Also, p-values between 0.05 to 0.1 were misinterpreted as statistical trends more than 60% of the time, a result that confirms researcher bias and highlights that spin continues to be a major problem in science. ^20^ Worryingly, the number of papers that included undefined summary statistics increased from 12.5% of papers in 2015 to 22.6% of papers in 2019-2020. Such high rates of spin and poor scientific reporting are worrisome. Overall, results of the present audit show that, at least in this journal, highlighting poor reporting practices does nothing to remedy the problem.

In 2016 and again in 2018, the American Physiological Society introduced several required or strongly encouraged reporting practices. Our results indicate that easy-to-implement required reporting practices were consistently adhered to in only a third of audited papers. Similarly, strongly encouraged reporting practices were consistently adhered to in only 12.0 to 25.0% of audited papers. While the introduction of new reporting requirements to improve the quality, transparency and reproducibility of published papers is well intentioned, our results indicate that they were not universally adopted and enforced at the Journal of Neurophysiology.

### Why does poor research reporting persist?

The above results raise an important question: Who is ultimately responsible for enforcing the reporting requirements outlined in a journal’s *Information for Authors*? The obvious answer is the journal itself, its staff and its editors. However, as highlighted by our own work and that of others, ^12^^–^^23^ staff and editors across several journals do not take on this responsibility. While it is possible that editors do not agree with reporting practices required by their journal and thus do not choose to enforce them, a more plausible explanation is that editors, like the rest of us, are overworked. ^44^ They do not have the time to ensure papers they handle comply with every aspect of their *Information for Authors.* In line with this possibility, personal communication with an Associate Editor at the Journal of Neurophysiology confirms that editors are not required to read in their entirety the papers they handle. A similar comment was shared by an Associate Editor of the European Journal of Applied Physiology, highlighting that this is not an isolated practice. In addition, some editors lack knowledge about reporting guidelines while others fear that authors will not submit to a journal renowned for its strict reporting guidelines. ^45^ In the same vein, journal staff likely do not have the time (or expertise) to review every paper with a fine tooth comb to ensure they comply with reporting requirements. Some journals, including the Journal of Neurophysiology, use SciScore ( https://sciscore.com/) to assess and score submitted manuscripts in an attempt to enhance the rigor and reproducibility of published papers. Unfortunately, these tools are far from perfect and their output requires expert review. Moreover, the items assessed by SciScore at the Journal of Neurophysiology do not address any of the items in the present audit. Thus, despite being the obvious choice to enforce their own reporting requirements, many journals are not currently enforcing compliance with their *Information for Authors.*

Who else might be responsible for this enforcement? Reviewers? Reviewers are expected to focus on the science, not compliance with reporting requirements. Having said this, several journals require reviewers to complete a series of tick-box questions to indicate whether or not the paper they are reviewing complies with key reporting practices. At the Journal of Neurophysiology, reviewers are required to answer the following questions:’Should any bar graphs be presented as box-whisker plots or dot-whisker plots, instead of bar graphs, to enhance clarity?’,’Do bar graphs show individual data values?’, and, with regards to figure legends,’Do they include details for each individual experiment (sample number (n), sex, statistics test)?’. Based on the results of the present audit, this approach does not seem effective.

What about authors? Authors are expected to read a journal’s *Information for Authors* when preparing their manuscript. However, *Information for Authors* can be lengthy, convoluted and difficult to navigate, with each journal having their own bespoke version. Authors may not follow the *Information for Authors* to save time. Since the Journal of Neurophysiology has a rejection rate of ∼ 55%, ^46^ authors may not feel it is worth their time to comply with all of the requirements and recommendations in the *Information for Authors.* This is especially true when authors can peruse the current issue of the Journal of Neurophysiology and see that reporting requirements are not enforced. Authors already have to deal with high rejection rates, pedantic submission processes and tedious formatting requirements (at submission or acceptance) that amount to free labour for journals and publishers. ^47^ To pass the responsibility of compliance with *Information for Authors* to the authors would be taking further advantage of the skewed power dynamic that exists between journals, publishers and authors. Having said this, a simple checklist related to compliance with reporting requirements could be required at submission or acceptance. However, someone would still need to verify and enforce results from these checklists. As mentioned above, a similar checklist is already in place at the Journal of Neurophysiology for reviewers, and it appears to be largely ineffective. Thus, it is unclear whether introducing a checklist for authors to complete would fare any better.

### Possible solutions to improve research reporting

A possible solution would be for editors to be assigned a small team of junior editors. These junior editors would assess papers that go out for review for compliance with reporting practices outlined in the *Information for Authors.* Such an approach, if adhered to and properly enforced, could ensure required and recommended reporting practices become the norm rather than the exception. In fact, some American Physiological Society Journals have introduced an Early Career Editorial Fellowship Program. ^48^ This is a great initiative, and the scope of the program could be broadened to tackle the tasks mentioned above. Unfortunately, few *Information for Authors* address good reporting practices ^49^; an issue that would need to be addressed for this solution to work.

Another possible solution would be for journals to employ additional staff with the experience and expertise to review submitted manuscripts. It may be that software tools can be used in the first instance, with journal staff carefully reviewing papers only once they are likely to be accepted. The obvious problem with this solution is that some journals are run on small budgets, while other journals are run by large profit-driven corporations that are unlikely to support the creation of such positions.

In healthcare, published guidelines do not automatically translate into clinical practice. ^50^ This type of behavioural change must be supported by multiple strategies that target all aspects of a system. ^51^ A similar approach may be required to change adherence to reporting guidelines in academic journals.

### Limitations

We acknowledge there are limitations to conducting an audit of papers published in a single journal. How generalisable are our results? While the present study can be viewed as a case study, its results are, for the most part, in line with two previous large-scale audits: one that involved over 750 papers published in the Journal of Physiology and the British Journal of Pharmacology, ^32^ the other that involved nearly 500 papers published by a medical research institute across dozens of journals and disciplines. ^34^ Our results are also in line with other recent reports of poor scientific reporting, ^17^^–^^23^^,^^52^ including an audit where 61% of published COVID-19 studies used incorrect statistical methods. ^53^ Although the present audit focused on a single journal, we did not set out to target the Journal of Neurophysiology *per se.* Our previous 2015 audit ^33^ and the introduction of new reporting requirements presented a unique opportunity to answer the research questions tackled here. Whether the present results are applicable to other journals remains to be determined.

To reduce the bias in data extraction to a minimum, it would preferable if future audits were performed in duplicate. Two investigators would audit each paper and any disagreements would be resolved in discussion with a third investigator.

## Conclusions

Accurate and unbiased scientific reporting is essential. However, to assume authors will always comply with reporting requirements and apply research best practices is idealistic. Similarly, to assume that editors and reviewers – who, at other times, are also authors – will rise to the occasion without stronger incentives (or compensation) is also unrealistic. Education does not lead to substantial change, ^32^ nor does updating *Information for Authors* or publicly highlighting poor reporting practices, as this audit illustrates. Novel solutions that acknowledge and address the complexity of the scientific enterprise and the various incentives that are at play are sorely needed.

## Data Availability

Figshare: Poor statistical reporting, inadequate data presentation and spin persist despite Journal awareness and updated Information for Authors. https://doi.org/10.6084/m9.figshare.c.6920920.v1. ^54^ This project contains the following underlying data:
-
**Audit results.** Spreadsheet containing all audit data. Also included are summary statistics for each audit item (overall, 2019, 2020). -
**Extract from Information for Authors.** Extract from the American Physiological Society *Information for Authors.* Specifically, the sections on 1) Promoting Transparent Reporting in APS Publications to Enhance Data Reproducibility; 2) Data presentation, and 3) Experimental Details to Report in Your Manuscript. This project contains the following extended data:
-
**Scoring manual.** Manual that explains each audit item. Key terms and the scope of each audit item are defined, and possible answers specified. Examples are also provided. -
**PRISMA Checklists.** Completed PRISMA Checklist and PRISMA Abstract Checklist. Data are available under the terms of the https://creativecommons.org/licenses/by/4.0/Creative Commons Attribution 4.0 International license (CC-BY 4.0).
