# Supplementary material for: Poor statistical reporting, inadequate data presentation and spin persist despite Journal awareness and updated Information for Authors
Source: F1000Res. 2023 Nov 20;12:1483. [Version 1] doi: 10.12688/f1000research.142841.1 (PMC10905014; doi:10.12688/f1000research.142841.1)
Supplement: Peer review history

# Reviewer report: Reviewer response for version 1

**DOI:** 10.5256/f1000research.156438.r236603

**Published:** 2024-02-28

**Authors:** Christopher McCrum

**Affiliations:** 1 Maastricht University Medical Centre, Maastricht, The Netherlands

The manuscript presents the results of an audit of specific reporting practices in recent issues of a journal and compares these with previous audits, in order to evaluate if changes in author guidelines and requirements have influenced practice. This is an interesting and important manuscript and while only conducted for one journal, the results are in line with many previous audits at other journals. The discussion highlights some of the important issues in attempting to improve these practices. The manuscript is clearly written and reported and the associated data and resources are accessible (except for the PRISMA material mentioned). I have no major comments that the authors need to address. I have a few general comments for the authors to consider.

 Some phrasing in the manuscript indicates that causality might be determinable with the current auditing approach (e.g., "We provide evidence that publicly highlighting poor reporting practices in a journal does not lead to improvements.") but I think only association could be determined by observing changes in reporting following journal guideline changes. Consider rephrasing some sections of the discussion on this point. 

 The approach and many results are comparable to results on sample size calculation reporting in two recent studies that I have been involved in. In those papers, journals that explicitly required justification of the sample size used also only saw adherence in the ~30-40% range (1, 2). The current manuscript clearly confirms the findings that including requirements in author guidelines are not sufficient to drastically improve reporting. Therefore, I wonder if one additional/expanded recommendation in the discussion that could be made is the inclusion of a specific question in the peer review process (either for editors or reviewers), expanding on the current checkbox approach that the authors discuss, on whether or not the manuscript adheres to a stated journal requirement and where in the article this information is provided. Acceptance of the manuscript for publication could be dependent on a positive response on this item (I think often not the case). This would not require any additional staff or budget and would not necessarily require additional expertise from the reviewers, since they only have to be able to identify the presence of the information, not its validity. I think the discussed concern about journal appearing to "strict" in their requirements lacks evidence, since some of the most prestigious medical journals tend to have extremely specific and detailed requirements for their manuscripts (not just statistical reporting) and still attract submissions. I understand it is not the aim of the current manuscript to provide solutions, but the authors could expand on their thoughts for future solutions to explore.

Is the case presented with sufficient detail to be useful for teaching or other practitioners?

Yes

Is the work clearly and accurately presented and does it cite the current literature?

Partly

If applicable, is the statistical analysis and its interpretation appropriate?

Not applicable

Are all the source data underlying the results available to ensure full reproducibility?

Yes

Are the conclusions drawn adequately supported by the results?

Partly

Is the background of the case’s history and progression described in sufficient detail?

Yes

Reviewer Expertise:

Human Movement Science, Meta-Science

I confirm that I have read this submission and believe that I have an appropriate level of expertise to confirm that it is of an acceptable scientific standard.

## References

[1] : Nature of Our Literature. *Communications in Kinesiology* .2021;1(3) : 10.51224/cik.v1i3.43 10.51224/cik.v1i3.43

[2] : Sample size justifications in Gait & Posture. *Gait Posture* .2022;92: 10.1016/j.gaitpost.2021.12.010 333-337 10.1016/j.gaitpost.2021.12.010 34920357

# Reviewer report: Reviewer response for version 1

**DOI:** 10.5256/f1000research.156438.r229985

**Published:** 2024-02-09

**Authors:** Clarissa F. D. Carneiro

**Affiliations:** 1 Berlin Institute of Health at Charité, QUEST Center for Responsible Research,, Berlin, Germany

The paper presents the results of an assessment of the quality of statistical reporting in publications from the Journal of Neurophysiology (2019-2020). The paper is very concise and direct in its presentation of the study, what makes it easy and pleasant to read. The down side of this conciseness is that we depend on consulting the supplementary materials to understand some details of the table (e.g., why do the denominators change in different items) and some bits of the discussion are quite vague. I list a few suggestions and open questions below, but how much to deepen the discussion and description of the methods and data in the main manuscript text can be a matter of personal preference. In any case, the data and other additional materials available are very well organized and easy to consult and re-use.

- The main concern I have is with the use of causal language. As this is an observational study, causality cannot be inferred but is implied in some sections of the text. To highlight just one example, the first sentence of the discussion (“We provide evidence that publicly highlighting poor reporting practices in a journal does not lead to improvements.”) implies a causality link but the correlation would be better described as “We provide evidence that publicly highlighting poor reporting practices in a journal is not associated with improvements.”.
- The main open question I have is if the creation and updating of the *Information to authors* by the publisher was accompanied by similar updates in the guides/instructions to reviewers or any training material to editors. Reviewers and editors can have lots of different interpretations about their roles (e.g.: Glonti *et al.*, 2019 ^1^; Glonti and Hren, 2018 ^2^), and reviewers assessing papers for different journals or publishers may not be aware of specific editorial policies or requirements.
- The different ways that spin is referred to throughout the text can be confusing for readers who are not familiar with the concept. Spin can be much more complex and difficult to detect/assess than simply describing a p-value close to 0.05 as a trend of significance, and this is only superficially implied throughout this paper. If the authors prefer not to go into detail of definitions within the current paper, listing references 14 and 15 next to the definitions of spin could help guide the reader.  
- A trivial question that emerged as reading the paper was if the Journal of Neurophysiology is published by the American Physiological Society. This could be an obvious link to physiologists and can easily be solved with a quick search online, but might be worth explicitly stating in the introduction for a better logic flow.
- Regarding the use of checklists as mentioned in the discussion, there is further evidence suggesting they are not very effective in improving quality of reporting which would make the argument against checklists for authors stronger (e.g. Hair *et al.*, 2019 ^3^). In addition, these tasks if unchecked can quickly compound with other redundant or meaningless ones contributing to a negative impact and/or perception of responsible research practices (e.g., Cochran *et al.*, 2024 ^4^).
- I personally agree with the suggestion that the journal staff should be primarily responsible for reviewing quality of reporting, but also understand the limitations described. Do the authors believe the problems of small budgets and profit-driven motivations could be reduced in large scientific society publishers, like the APS? It could be an interesting addition to the discussion.
- Regarding behavioral changes, I’d also be curious to read more on the authors views on approaches from clinical practice that could be translated to the publication system. There are other models of behavioral change being proposed for open/responsible research practices that might also be worth discussing (e.g.: Norris and O'Connor, 2019 ^5^; Robson et al., 2021 ^6^; https://www.ncbi.nlm.nih.gov/books/NBK556627/).
- One final small question, when assessing data availability: if a repository link or ID is given, did the assessors check that it worked? Or just presenting a link was sufficient to score “yes”? This is a potential limitation (e.g.: Federer (2022 ^7^).  

Is the case presented with sufficient detail to be useful for teaching or other practitioners?

Yes

Is the work clearly and accurately presented and does it cite the current literature?

Partly

If applicable, is the statistical analysis and its interpretation appropriate?

Yes

Are all the source data underlying the results available to ensure full reproducibility?

Yes

Are the conclusions drawn adequately supported by the results?

Partly

Is the background of the case’s history and progression described in sufficient detail?

Yes

Reviewer Expertise:

meta-research, scholarly publication, open, responsible and reproducible research practices

I confirm that I have read this submission and believe that I have an appropriate level of expertise to confirm that it is of an acceptable scientific standard, however I have significant reservations, as outlined above.

## References

[1] : A scoping review on the roles and tasks of peer reviewers in the manuscript review process in biomedical journals. *BMC Med* .2019;17(1) : 10.1186/s12916-019-1347-0 118 10.1186/s12916-019-1347-0 31217033 PMC6585141

[2] : Editors' perspectives on the peer-review process in biomedical journals: protocol for a qualitative study. *BMJ Open* .2018;8(10) : 10.1136/bmjopen-2017-020568 e020568 10.1136/bmjopen-2017-020568 30341111 PMC6196803

[3] : A randomised controlled trial of an Intervention to Improve Compliance with the ARRIVE guidelines (IICARus). *Res Integr Peer Rev* .2019;4: 10.1186/s41073-019-0069-3 12 10.1186/s41073-019-0069-3 31205756 PMC6560728

[4] : Cell cycle specific, differentially tagged ribosomal proteins to measure phase specific transcriptomes from asynchronously cycling cells. *Sci Rep* .2024;14(1) : 10.1038/s41598-024-52085-5 1623 10.1038/s41598-024-52085-5 38238470 PMC10796924

[5] : Science as behaviour: Using a behaviour change approach to increase uptake of open science. *Psychology & Health* .2019;34(12) : 10.1080/08870446.2019.1679373 1397-1406 10.1080/08870446.2019.1679373 31661325

[6] : Promoting Open Science: A Holistic Approach to Changing Behaviour. *Collabra: Psychology* .2021;7(1) : 10.1525/collabra.30137 10.1525/collabra.30137

[7] : Long-term availability of data associated with articles in PLOS ONE. *PLoS One* .2022;17(8) : 10.1371/journal.pone.0272845 e0272845 10.1371/journal.pone.0272845 36001577 PMC9401135

# Reviewer report: Reviewer response for version 1

**DOI:** 10.5256/f1000research.156438.r229987

**Published:** 2024-02-09

**Authors:** Daniel J. Dunleavy

**Affiliations:** 1 Florida State University, Tallahassee, Florida, USA

The authors have written a case study about the statistical reporting practices of articles published in the J *ournal of Neurophysiology (JNP) *for the years 2019 and 2020. The study supports a wide ranging body of work, which finds suboptimal, if not erroneous, reporting of statistical methods and results across the biomedical and social sciences. (Pocock *et al.*, 1987 ^1^; Nuijten *et al.*, 2016 ^2^; Dunleavy, 2020 ^3^; Ziliak, 2004 ^4^).

 While there have been calls and initiatives to improve the reporting of quantitative studies within the field of physiology - for example, editorials published by the Journal of Physiology and British Journal of Pharmacology; efforts by the American Physiological Society to revise its "Information for Authors" pages - little is known about their impact. 

 To explore this, the authors audited 580 papers published in JNP, published after changes to the journal's Information for Authors page had been put into effect. Many of the reporting practices examined remained unchanged or worsened between the two timepoints.

 Overall, I found the paper to be of interest, its methods relatively sound, and its conclusions reasonable.

 I hope the following comments, questions, and suggestions help strengthen the overall quality of the manuscript. Additionally, please be advised that any and all references included in this review are suggestive and not meant to be viewed as compulsory.

**Data Availability and Integrity:**

 1a. I can confirm that I was able to access the spreadsheet containing audit results for each article. Further, I selected a handful of results and was able to identify and locate the corresponding article using the "paper" variable.

 1b. After opening several papers, I was able to verify a handful of the coded results.

 2a. I was also able to locate and understand the scoring manual and locate a copy of the "Information for Authors" page.

 2b. However, I was not able to locate the PRISMA Checklist associated with the current study.

**Reporting of Methods and Results:**

 3. The authors indicated that there were 580 published papers audited in the study. Personally, I'd like to see more context about the total number of papers published in the journal, for the two years (e.g., including a breakdown of articles excluded and their type - editorials, commentaries, errata. etc.).

 4. The authors state, "Two investigators would audit each paper and any disagreements would be resolved in discussion with a third investigator." I'd like to see more information about how common disagreements were, even if they were able to be fully resolved. This would permit the reader a clearer understanding about whether certain variables/items were more difficult to code than others.

**Miscellaneous:**

 5. The authors do a good job acknowledging the limitations of their study - primarily that it focuses on a single journal, during a short period of time. However, the title of this case study does not make this clear. If possible, I'd recommend changing the title of the paper to something more descriptive, like:

 "Poor statistical reporting, inadequate data presentation and spin persist: A study of papers published in the Journal of Neurophysiology, 2019-2020"

 6. To the extent that preprints are used in this field, the authors might consider exploring what changes are made between preprint version of manuscripts and their published versions. This might give some indication into how peer review and editorial oversight at JNP impacts the content and reporting of papers. Some interesting work is being done by Mario Malički and colleagues (Malički *et al.*, 2024 ^5^)

Is the case presented with sufficient detail to be useful for teaching or other practitioners?

Yes

Is the work clearly and accurately presented and does it cite the current literature?

Yes

If applicable, is the statistical analysis and its interpretation appropriate?

Yes

Are all the source data underlying the results available to ensure full reproducibility?

Yes

Are the conclusions drawn adequately supported by the results?

Partly

Is the background of the case’s history and progression described in sufficient detail?

Yes

Reviewer Expertise:

I am an academic social worker by training (PhD) with a record of publishing on issues related to meta-science, open science, peer review, and other issues in scholarly publishing.

I confirm that I have read this submission and believe that I have an appropriate level of expertise to confirm that it is of an acceptable scientific standard.

## References

[1] : Statistical problems in the reporting of clinical trials. A survey of three medical journals. *N Engl J Med* .1987;317(7) : 10.1056/NEJM198708133170706 426-32 10.1056/NEJM198708133170706 3614286

[2] : The prevalence of statistical reporting errors in psychology (1985-2013). *Behav Res Methods* .2016;48(4) : 10.3758/s13428-015-0664-2 1205-1226 10.3758/s13428-015-0664-2 26497820 PMC5101263

[3] : Appraising Contemporary Social Work Research: Meta-Research on Statistical Reporting, Statistical Power, and Evidential Value.2020; 10.31237/osf.io/f2c7b 10.31237/osf.io/f2c7b

[4] : Size matters: the standard error of regressions in the American Economic Review. *The Journal of Socio-Economics* .2004;33(5) : 10.1016/j.socec.2004.09.024 527-546 10.1016/j.socec.2004.09.024

[5] : Comparison of Reports of Epidemiology Studies Posted as bioRxiv Preprints and Published in Peer Reviewed Journals. *International Congress on Peer Review and Scientific Publication* .2024; Reference source

# Reviewer report: Reviewer response for version 1

**DOI:** 10.5256/f1000research.156438.r226880

**Published:** 2023-12-14

**Authors:** Ulf Kahlert

**Affiliations:** 1 University Clinic for General-, Visceral-, Vascular- and Transplantation Surgery, Medical Faculty University Hospital, Magdeburg,, Germany

Héroux and colleagues conducted a tedious work. Comparing pre- and post audit papers published by an investigated journal incorporates comparing of changes in behavior of authors and the journal. This setup is ideal for comparison and worth investigating.  The presented results from this sector of research continues to be worrisome.

 The required reporting items as defined in the paper differ from the journal information for authors. Journal states that the statistical test and number of animals/models per group shall be stated in the material and methods section. Although I agree to Héroux and colleagues that stating these details in the figure legend is necessary to “sufficiently describe the figure on their own, without reference to the main text (as stated in the Information for authors of the journal homepage), the scoring for reporting completeness might be different. On the journal homepage, for the figure legends, regarding statistics it says: “Define all statistical symbols and abbreviations”.

 I think the title of the paper shall indicate to the main conclusion drawn: introduction of recommendations/guidelines have not sufficient penetration to all authors. This may draw more attention,  as lot of people focus on recommendations - which is good - but the impact might be overestimated.

 Suggested aspects to be changed: What responsibility could the authors’ home institution play? Shall publication with using a given affiliations imply control measures of written results before submission. Also grant agencies: shall there be a paragraph to provide published evidence that a lab of a given applicant enforces measures / adhere to complete reporting? The society debates about sustainability. Only reproducible science has high chance for sustaining.

Is the case presented with sufficient detail to be useful for teaching or other practitioners?

Yes

Is the work clearly and accurately presented and does it cite the current literature?

Partly

If applicable, is the statistical analysis and its interpretation appropriate?

I cannot comment. A qualified statistician is required.

Are all the source data underlying the results available to ensure full reproducibility?

Yes

Are the conclusions drawn adequately supported by the results?

Yes

Is the background of the case’s history and progression described in sufficient detail?

Yes

Reviewer Expertise:

cancer research

I confirm that I have read this submission and believe that I have an appropriate level of expertise to confirm that it is of an acceptable scientific standard, however I have significant reservations, as outlined above.
